# Trauma‐informed bequeathed body donor meeting sessions: A guide for creating a supportive and humanistic anatomy laboratory

**DOI:** 10.1002/ase.70177

**Published:** 2025-12-25

**Authors:** Bryn Bhalerao, Catherine Belling, Angelique N. Dueñas, Charys M. Martin, Andrew M. Deweyert

**Affiliations:** ^1^ Department of Anatomy and Cell Biology, Schulich School of Medicine and Dentistry Western University London Ontario Canada; ^2^ Department of Medical Education, Feinberg School of Medicine Northwestern University Chicago Illinois USA

**Keywords:** anatomy education, dissection and Prosection curricula, donor meeting session, emotional preparedness, gross anatomy laboratory, humanism in medical education, inclusive curriculum design, medical student wellness, peer support, person‐centered learning, professional identity formation, reflective practice, trauma‐informed education, undergraduate medical education

## Abstract

Anatomy educators are increasingly seeking approaches that honor the humanity of body donors while supporting learners through their first encounters in the gross anatomy lab. We describe a comprehensive donor meeting session, implemented in both dissection and prosection curricula at two North American medical schools, that prepares students to engage with donors before formal content–based learning begins. Grounded in the six principles of trauma‐informed practice—safety; trustworthiness and transparency; peer support; collaboration; empowerment; and inclusivity—the session prioritizes relational and humanistic engagement over technical preparation alone. Learner reflections and faculty observations indicate that this model promotes emotional readiness, mitigates distress, and cultivates gratitude and humanism. We offer this framework for anatomy educators aiming to create supportive, donor‐centered learning environments that shape both professional identity and future patient care.

## INTRODUCTION

The gross anatomy laboratory (lab) is a critical component of medical school curricula as an environment for learning human anatomy. Additionally, this learning environment also introduces foundational professional skills required for health care providers, aligning with calls to integrate professionalism and ethics into anatomy education.^1^ These skills include collaboration, peer support, personal empowerment, respect, empathy, and emotional regulation. The foundations of these skills are interwoven with the knowledge acquisition in the lab setting often through group work or observation and interaction with bequeathed body donors. The experience of working in a gross anatomy lab is a significant life experience perceived differently by different learners due to the nature and novelty of the environment.^2^, ^3^, ^4^ Initial experiences in the gross anatomy lab often represent students’ first direct encounter with a deceased person, an experience that can evoke a range of complex and sometimes difficult emotions.^5^ Thus, the anatomy lab can become an arena for self‐discovery as students encounter and cope with feelings associated with death, dying, and bereavement.^6^ Traditionally, students were encouraged to adopt a stance of detached concern, allowing them to set aside their emotional responses in order to concentrate on anatomical knowledge, technical skills, and clinical application.^5^ Recent developments in the field challenge this paradigm and reframe the exposure as an opportunity to develop humanistic skills also crucial for future health care providers. For example, one relevant coping mechanism is adopting either a “specimen‐minded” view or a “person‐minded” view of a donor, whereby the donor is viewed as either a specimen or a person.^7^ However, there is also opportunity to practice other psychological coping strategies, such as “toggling” between different mindsets, detached concern, or utilizing healthy compartmentalization, which are all important for future clinical practice.^8^, ^9^

The opportunity and importance of developing professional skills and coping mechanisms are relevant regardless of the anatomy curriculum, whether dissection‐ or prosection‐based.^10^ To support students in navigating their first encounters with body donors, most medical schools implement some form of preparatory session.^11^ These sessions vary widely across institutions but often focus on the logistics of working and learning in the gross anatomy lab and do not consistently include structured opportunities for reflection.^12^ Reflection is more commonly reserved for memorial services held after the completion of the course, bringing together students, instructors, and donor families. Other innovative approaches have sought to address the emotional impact earlier in training: for example, Bertman and Marks implemented a separate course on death and dying that included a predissection introductory session, which decreased hesitation about dissection and encouraged empathy in preclinical students^6^; Souza and colleagues developed the “Cadaver as First Teacher” modules to foster ethics and humanism^13^; Bohl and colleagues used prerecorded donor interviews to strengthen students' sense of personal connection to their donors^14^; and Guo and colleagues conducted a precourse gratitude ceremony with donors' families that reduced apprehension and promoted humane attitudes toward both donors and patients.^5^ Collectively, these studies demonstrate that structured preparation can foster empathy, respect, and humanism in anatomy labs. Building on this foundation, our work takes an additional step by intentionally promoting not only professional skills but also student wellness, implementing a trauma‐informed approach to support learners holistically in the anatomy lab.

At both Western University's Schulich School of Medicine & Dentistry (London, ON, Canada) and Northwestern University's Feinberg School of Medicine (Chicago, IL, USA), gross anatomy is a foundational part of the medical curriculum. Although Schulich uses whole‐body dissection and Feinberg employs a prosection‐based approach, where learners study professionally dissected human tissue from bequeathed donors, students at both schools share the profound opportunity to learn from individuals who have donated their bodies through Western's Body Bequeathal Program and the Anatomical Gift Association of Illinois. In recent years, anatomy faculty at both institutions have observed a noticeable shift in how students respond, both physically and emotionally, during their first encounters in the gross anatomy lab. Increasingly, students have needed to step away from the lab during the initial session and have found it difficult to return and re‐engage. This emotional difficulty, while entirely valid, presents a dual challenge. First, it affects student learning and wellness. Second, it places instructors in the position of providing emotional support, which they may not be qualified to provide, and removes them temporarily from their primary responsibility, which is to guide students through complex anatomical content.

Concurrently, there have been broader shifts in medical education, where more holistic, person‐centered approaches are increasingly shaping how students relate to the human body and bequeathed donors.^15^ While this evolution in the field speaks to the growing sense of humanism and respect students bring into the lab, the patterns observed in the Schulich and Feinberg medical students also signal the need for *dedicated* resources and support that prepare students emotionally, so they can fully engage with the learning experience while honoring the generosity of those who have donated their bodies to science. Ghosh and Kumar^16^ highlighted the importance of these interventions in fostering humanistic skills within anatomy labs early in physicians' training.^16^ This sentiment adds to findings by Rizzolo,^17^ who describes the anatomy lab as an invaluable setting for cultivating professional skills essential to patient care, while also emphasizing the importance of allowing students time and space to appreciate its significance without the added pressure of academic performance.^17^ McDaniel^18^ and colleagues also emphasize the importance of trauma‐informed medical education and its relevance to providing support to students through the transition into the anatomy lab.^18^ By recognizing both the emotional weight of anatomical learning and the educational role of anatomy faculty, we can better support students in developing into compassionate clinicians, without compromising the integrity of lab‐based teaching. Recognizing the responsibility to support learners through this formative experience, we implemented a comprehensive, trauma‐informed approach to donor introductions that both supports student wellness and reinforces a humanistic curriculum.^19^ This descriptive article illustrates how intentionally designed sessions that encourage self‐reflection can foster skill development, support student wellness, and contribute to the formation of professional identity for health care students learning in a gross anatomy lab.

## DONOR MEETING SESSION DESCRIPTIONS

Prior to 2021, both schools’ undergraduate medical anatomy curricula included an “Introduction to Anatomy” session to cover gross anatomy lab protocols and expectations for the dissection and prosection labs. However, there were no formal lab or donor‐related introductions before their first gross anatomy lab. Students’ first encounter with bequeathed body donors coincided with their first content lab in which they were expected to accomplish the learning objectives. There were no donor “meetings” or opportunities for self‐reflection included in the curricula.

The motivation for this curricular change was to create a lab environment that supports both students and instructors and fosters reflection through a trauma‐informed lens. This approach is grounded in six key principles: safety, trustworthiness and transparency, peer support, collaboration, empowerment, and inclusivity as articulated by the Substance Abuse and Mental Health Services Administration (SAMHSA).^19^ While elements of our donor meeting session share commonalities with previously described practices—such as ethics and reflection modules,^13^ prelaboratory gratitude ceremonies,^5^ respectful language initiatives,^15^, ^20^ and memorial activities^6^—our approach is distinct in both conceptual framework and implementation. This framework is guided not only by content and language choices but also the physical environment, facilitation style, and follow‐up support, aiming to anticipate and mitigate potential triggers for all participants. These sessions respond to what students most want to know about their body donors^21^ by incorporating family‐provided personal histories and medical histories, and by unveiling the whole donor before dissection begins. Together, these practices restore the donor's identity,^22^ through both narrative and physical presence, before the body is dissected for anatomical study, reducing the risk of objectification and reinforcing a humanistic perspective. Additionally, structured reflective activities combined with facilitated small‐group discussions provide learners with personalization and emotional processing tools. Lab managers at both institutions ensure that students are not exposed to loved ones who may have donated through cross‐referencing last names and responding to student disclosures.

In October 2021, two medical educators (authors AND and AMD) and a medical ethicist (author CB) implemented a donor meeting session at Feinberg. In September 2023, the donor meeting session was adapted by the medical anatomy educators at Schulich (authors AMD and CM) for their dissection‐based curriculum. It is important to note that these sessions were conducted at least one week in advance of the commencement of dissection/prosection‐based lab sessions that required learning anatomy‐related content from donor specimens.

### Prosection‐based donor meeting session

#### In‐class session

**FIGURE 1:**
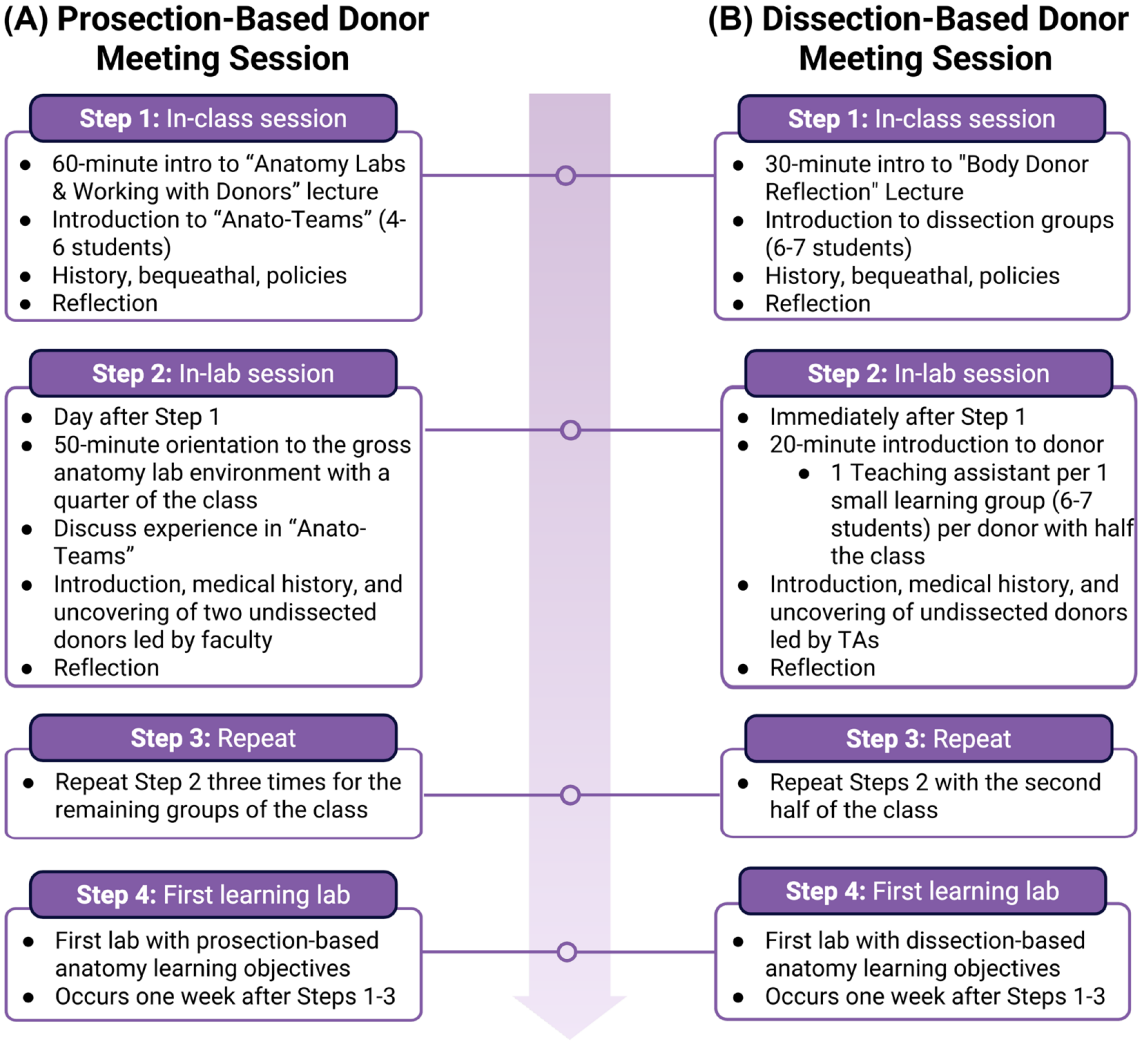
Overview of donor meeting sessions at two medical schools. Overview of a (A) Prosection‐based donor meeting session at Feinberg School of Medicine and a (B) dissection‐based donor meeting session at the Schulich School of Medicine and Dentistry. The panels provide a visual demonstration of the order of steps for each of the donor meeting sessions associated with the prosection‐based and dissection‐based curriculum. This figure was created using BioRender.

At Feinberg, anatomy was first introduced early in the undergraduate medical education curriculum during “Foundations 2”. One of the first anatomy sessions in this module was a 60‐min required in‐person lecture entitled “Intro to Anatomy Labs & Working with Donors” that was conducted prior to the commencement of prosection‐based laboratory sessions (Figure 1A). The in‐person session included informing students about the practical and logistical elements of learning from prosections; such information included how labs are organized, appropriate attire, and expectations for working in the gross anatomy lab. In efforts to promote the teamwork and communication skills that were cited as important facets of dissection‐based curriculum, this session also introduced the students to their “Anato‐Teams,” small groups of 4–6 students with whom they work throughout their entire anatomy lab curriculum.

The session then transitioned from practical to more personal and reflective elements, starting with the donors. There was a brief description of the donor bequeathal (all donors at Feinberg are obtained through the Anatomical Gift Association of Illinois), and the process of donation was briefly framed in the historical context of body donation in medical education. Of particular importance to the Feinberg community, the intersection and interplay of body donation, marginalization, and inclusionary practices are discussed. Since 2023, this element has included a student‐developed portion that focuses on a brief racial history of human tissue use for medical education. Students were then invited to join the planning committee for the end of the year body donor memorial ceremony before the final portion of the session, led by a medical ethicist (author CB). This closing segment encouraged learners to reflect on what they hoped to learn beyond anatomy content and to view their emotional responses to the donors as a valuable step in becoming a physician. Students were asked to briefly write about their expectations for their first lab experience, after which they were provided with contact information for university counseling services.

#### In‐lab session

The day after the in‐person session, the entire class participated in an introductory laboratory session. To accommodate class size (*n* = 140–160), the class was divided into four smaller groups (*n* = 40 maximum); all prosection lab sessions were repeated four times to accommodate each of these smaller groups. Similarly, the 50‐min in‐lab session was repeated four times with each of these groups (Figure 1A).

The in‐lab component began by orienting students to the laboratory environment and the practical aspects of working in a prosection lab setting. This introduction included informing and orienting students to the layout and resources of the lab, as well as having students briefly meet in their “Anato‐Teams” to discuss past anatomy experiences, team dynamics, and anticipated challenges and opportunities in anatomy education. Students were then encouraged to gather around two undissected donors. The donor meeting began with reminders about available supports, such as seating in the lab, faculty support if students needed to step out, and access to additional resources. The faculty then introduced the still‐covered donors by sharing medical histories, including age, diagnoses, cause of death, and any personal details provided. Donors were uncovered in synchronicity by two faculty members. After the donors were uncovered, students were encouraged to recall their anticipatory reflections and compare these with their immediate responses to being present in the lab in the presence of the donors. They had a brief period of time to ask any questions, palpate the donors (if interested), or share reflections. Students were then invited to help cover and close the donor coverings, to model best practice for donor care after any time spent learning in the space. Students were reminded of supportive services as they were being instructed on the clean‐up steps required to leave the anatomy lab.

### Dissection‐based donor meeting session

#### In class session

At Schulich, anatomy was first introduced in the first course of the undergraduate medical education curriculum in “Foundations of Medicine.” A week *prior* to the first dissection‐based learning lab, medical students attended a 90‐min Body Donor Reflection Session that began with a 30‐min introductory lecture (Figure 1B). The lecture contained an overview of the history of anatomical dissection, Schulich's Body Bequeathal program, and an introduction to lab policies and procedures. The lecture focused on the dark history of anatomy, how far we have come, and where we still need to go. Respectful and ethical behavior expectations were also outlined with an emphasis on humanism, honoring donors' wishes and reflecting on the uniqueness of the experience. The lecture was followed by a reading of reflections about body donation provided by former students and family members of donors. Students were provided with a reflection framework to implement before, during, and after meeting their body donor. The in‐class session concluded by providing a list of general and Schulich‐specific wellness resources should they want to seek support throughout their experience with gross anatomy. Such resources included links to the Learners Experience Office, Western Mental Health Services, and Crisis Resources phone numbers. At the end of the academic year, students were encouraged to return to reflection on the experience of working with donors and to share their reflections during the annual spring body donor memorial service.

#### In‐lab session

In order to accommodate for the class size (*n* = 150 students), students were divided into two smaller groups (*n* = 75) and the in‐lab session was conducted twice, once for each group. For each undertaking, students were divided into their dissection groups of 5–7 students, with each dissection group having a dedicated teaching assistant. Running two separate in‐lab sessions was preferable to reduce the number of people in the lab at a time, making the environment quieter and more private, and to ensure that students received undivided attention and support from the instructional team. Each of the dissection groups gathered around their assigned, covered, undissected donor with their teaching assistant who led the 20‐min session (Figure 1B). Students were reminded of the resources available, which included chairs in the lab if they wished to sit, faculty available in the hall if they needed to exit the lab, and support services from a registered counselor. Next, the teaching assistants introduced the still‐covered donors through medical histories, such as age, diagnosed medical conditions, cause of death, and any provided personal histories. The instructor (author AMD) then signaled to the teaching assistants that it was time to proceed to uncover the donors. The complete uncovering of the donor was a step‐by‐step process with the speed being uniquely dictated by the comfort levels of each individual group. Throughout the uncovering process, students were invited to touch and interact with their donors and express their feelings about meeting their “first patients”. After a period of silent reflection, teaching assistants encouraged the students to engage in conversation about what they were feeling, thinking, and seeing for the first time. Teaching assistants were empowered to share their thoughts and experiences, provide information about the embalming process, answer questions, and remind students about the privilege of being entrusted to fulfill the donors' wishes. Finally, students were asked to help re‐cover their donors, if they were comfortable, as teaching assistants reiterated where to find supportive services.

## STUDENT‐PERCEIVED BENEFITS

The primary goals of implementing the donor meeting sessions were to foster supportive learning environments and align our curricula with the humanistic priorities in modern anatomical and medical education. To determine whether or not the sessions successfully met these goals, and to inform future iterations of the sessions, we engaged in curricular evaluation to learn about how the sessions impacted students. This section summarizes the student‐perceived outcomes collected from the quality assurance data through optional postsession written reflections and curricular evaluation data (nSchulich = 20, nFeinberg = 50). Overall, students in both programs indicated that their experience with the donor meeting session was positive. Student reflections from both institutions noted: a perceived sense of support, describing the session as a good transition, instilling a sense of humanism, and encouraging reflection.

Students mentioned that the session created an environment where they felt supported and had the space to experience the emotions of meeting donors. Students described feeling a sense of support from the instructional team and stated that despite feeling “*out of [their] comfort zone*” while entering the lab and meeting their donors for the first time, they appreciated that the session provided a safe space to experience the emotions associated with their first encounters. One student commented that the donor meeting session “*gave me an opportunity to process those emotions so I'm very appreciative of the opportunity*.” Another student commented, “*[the session] felt very respectful to the donors and to my mental and emotional well being*”.

Students also appreciated the donor meeting session as it acted as a good transition into the laboratory environment without the expectation of having to touch, dissect, or learn from the donors right away. They stated that the donor meeting session not only helped them transition into the laboratory environment but made them feel more prepared for the subsequent encounters when they were expected to learn and dissect. One student remarked that the session “*gave me the chance to get adjusted to the reality of working with cadaver donors so that I felt less overwhelmed during the first lab*”. Additionally, the “*short and fixed period of time*” helped reassure students. Another student reflected on the brief lab session saying “*I think priming everyone in lecture, then again during the lab was very effective and gave us time to reflect and mentally prepare ourselves for anatomy*”.

The students also explicitly stated that they perceived their experience with the donor meeting session to instill a sense of humanism. Students reported feeling a sense of deep gratitude for the gift of body donation and felt that the donor meeting session allowed them to recognize the privilege of working and learning from donors. One student stated that the session “*allowed an opportunity to reflect on the sacrifice and altruism of the donor*,” while another reported, “*I think it really added a human element to the process and emphasized the gratitude I should be feeling in the situation*.” Another student disclosed that they viewed the donor meeting session as an act of respect “*to[ward] the donor and their family to be able to reflect on the whole person before diving straight into anatomy*”. Aptly summarized by one student, they came to understand “*the reality that we are working with real people that led real lives*.”

Through their responses, students recognized the benefit of the donor meeting session to encourage reflection. Students remarked that although the experience of meeting their donors for the first time was difficult and “*hard to process emotionally*”, they still perceived it as a positive experience as it “*was a great opportunity to reflect*” and identify the feelings that their first encounters provoked. For example, one student stated, “*I expected not to be bothered at all when meeting the body donor*. *I was surprised at how uncomfortable I felt when we went into the anatomy lab … it left me feeling a little frustrated that day in a way I can't really explain*” while another student reported, “*I didn't feel any negative emotions; I felt grateful for the opportunity*”. Another student stated that the session “*helped establish the importance of these anatomy sessions and gave an opportunity to process emotions well in advance of actually starting dissections*”. The students also commented that the donor meeting session encouraged them to reflect on the expectations of their future as doctors and recognize the difficulties of the medical field. A student mentioned that “*The donor meeting allowed to fully comprehend the expectations and responsibilities that were handed to me as a [medical student], which I found to be both incredibly enlightening and inspiring*.”

## DISCUSSION AND SIGNIFICANCE

This session is designed to foster meaningful connection between students and donors, rooted in a trauma‐informed approach that acknowledges the emotional complexity of working with anatomical donors. By intentionally creating space for students to reflect, share, and honor the donors' humanity, we challenge traditional norms of anatomical education that often emphasize objectivity at the expense of emotional engagement. Our approach is comparable to practices at other institutions also aiming to emphasize the humanity of donors. Such work includes that of Lin et al.,^23^ which found that interaction with donors' family members, and occasionally even the donors before their passing, increased students' compassion and respect for their donors.^23^ The outcomes of this approach to nonanonymous donations ultimately fostered meaningful connections between students and their donors, which in turn helped students to cope with death‐related anxieties.^23^ Additionally, by providing students with video interviews of donors and communications with donors' family members, Ghosh and Kumar^16^ address the hidden curriculum and build empathy, compassion, and integrity in students as a shift to a more “patient‐centred” approach to anatomy.^16^ Although both of these approaches expose students to the identity of the donors, our approach has a similar emphasis on connection and humanity, while the identity of our donors remains anonymous.

Other notable work has been shown to help prepare students for the gross anatomy lab by developing strategies that aim to better support the emotional demands of entering the lab environment. McDaniel et al.^18^ promoted emotional resilience and coping strategies by implementing modules for faculty and students, to be completed prior to and throughout the anatomy course.^18^ These modules provided information about ethics in anatomy, reflection prompts, and available support resources; this trauma‐informed framework was determined to build collaboration and trust within the gross anatomy course.^18^ Shiozawa et al.^24^ also aimed to enhance emotional resilience through an optional experiential learning seminar.^24^ The small group seminars had guided questions to encourage peer‐to‐peer discussions on medial professionalism and reflection on the dissection room experience. The seminars helped students to better understand their coping mechanisms of death and dying throughout medical school and their careers.^24^ Similarly, our sessions are focused on supporting the emotional and professional development of our learners through working with human body donors.

Although our donor meeting sessions share similarities with previously described approaches, our work builds upon and further extends prior implementations of trauma‐informed frameworks, such as those described by McDaniel et al.,^18^ by applying the model in an explicit and comprehensive manner. A substantial body of evidence demonstrates that the gross anatomy lab can elicit emotional and physical reactions in learners that resemble symptoms of posttraumatic stress, particularly during early encounters with body donors.^18^, ^25^, ^26^ Such anticipatory anxiety and emotional distress may hinder students' capacity to engage fully with the learning environment and develop essential coping, anatomical, and professional skills.^27^ At the same time, emerging research shows that well‐designed preparatory programs can effectively reduce distress, support faster adjustment to the lab, and transform initial encounters with donors into opportunities for humanistic and professional growth.^28^ In response to this evidence, we concluded that a trauma‐informed introductory session was warranted within our anatomy curricula. We therefore frame our session using the six key principles of trauma‐informed practice, as outlined by SAMHSA—safety; trustworthiness and transparency; peer support; collaboration; empowerment; and attention to cultural, historical, and gender issues—to provide an intentional pedagogical foundation that prioritizes learner well‐being, fosters engagement, and supports the development of professional identity.^19^

### Safety

Safety is foundational to a trauma‐informed approach, and it was a core design element of this session. Safety refers not only to physical surroundings, but also to the interpersonal dynamics and emotional context that shape learning environments.^19^ One focus of this comprehensive donor meeting session was to create a more positive environment for everyone involved in the gross anatomy lab, including learners, the instructional team, support staff, and the donors. This session provided a designated space and time for students to meet and reflect on their donors before engaging in content‐based learning. By experiencing the emotional weight of entering the lab and encountering the bequeathed persons in a structured and reflective way, learners were able to process this significant moment without the simultaneous pressure to begin learning anatomy. This intentional sequencing promoted psychological safety, allowing students to be more present and emotionally regulated in future lab sessions. Learner reflections corroborated this benefit, with many reporting that they felt more emotionally prepared for the first content lab, having already been introduced to the lab environment and their donors. Instructors also observed a substantial decrease in learners requiring support or leaving the lab during initial laboratory sessions following implementation of the donor meeting. These changes reflect a safer and more emotionally sustainable learning environment. While anatomy faculty are experienced in managing the pedagogical aspects of gross anatomy, they may not have formal training in supporting learners through the emotional challenges of working with anatomical donors. As such, it was critical to involve professional student support services and resources accessible to learners, as an integrated part of the session. This holistic approach to lab orientation acknowledges that emotional safety is as essential to student success as physical safety and academic preparedness.

### Trustworthiness and transparency

Trustworthiness and transparency are essential to building an emotionally supportive and pedagogically sound learning environment. Trauma‐informed practice emphasizes clarity, consistency, and openness to build and maintain trust across all levels of interaction. In this case, trust between learners, instructors, and peers within their own developing sense of capability.^19^ In this session, transparency begins with a clear explanation of the session's purpose, format, and expectations. Learners are guided through the body bequeathal process, including how donors are accepted, cared for, and integrated into the curriculum. This openness demystifies the process and reassures learners that donors are ethically sourced. It also emphasizes that their work in the lab honors the donor's explicit gift to medical education. This transparency promotes ethical clarity and supports the development of a professional identity rooted in respect and gratitude.

Trust is further cultivated by fostering early and meaningful relationships between instructors and learners. The reflective tone of the donor meeting provides a rare opportunity for vulnerability and mentorship outside the usual academic dynamic. Instructors share their own reflections, model compassionate engagement, and participate in discussions alongside learners, helping to establish a culture of openness and care. This relational foundation aligns with findings from McDaniel et al.,^18^ whose trauma‐informed approach demonstrated that intentional transparency and shared emotional processing can build trust and collaboration among learners and faculty, ultimately supporting the development of emotional resilience.^18^ In our context, these same principles guide the donor meeting session and lay the groundwork for deeper mentorship and professional development throughout the curriculum. By embedding transparency into both logistical processes and interpersonal interactions, the session strengthens trust not only in the learning environment and instructional team, but also in learners' confidence in their own readiness to engage with the emotional and intellectual demands of anatomy education and their future career in health care.

Trauma‐informed principles of trust and transparency also have implications beyond the session itself, helping to strengthen the relationship between anatomy programs and the communities who support them through body donation. For example, in Taiwan, promoting connections between students, donors, and their family members helped to overcome traditional cultural reluctance toward body donation and resulted in a surplus of donors.^23^ This approach to humanize anatomy built trust within the community and simultaneously improved student well‐being and donor recruitment.^23^ Similar to the positive effects of memorial ceremonies, these introductory sessions (or ceremonies of sorts) are another example of the way in which schools emphasize donor respect and honoring body donation.^29^, ^30^, ^31^

### Peer support and collaboration

Peer support and collaboration are intertwined principles in a trauma‐informed approach. Peer support involves building trust, fostering connection, and creating space for learners to draw on their lived experiences and shared emotional responses. Collaboration emphasizes that all individuals, learners, instructors, support staff, and donors have a role to play and that power differences should be acknowledged and leveled where possible.^19^ Together, these principles promote mutual respect and co‐created learning environments grounded in empathy. Our sessions inherently require and build peer support and collaboration in the first weeks of our undergraduate medical curricula; this aligns with the perspectives in the medical education field to prioritize the direct incorporation of teamwork training into medical education as early as possible.^32^ In this session, learners were encouraged to express their feelings and reflections, drawing from their own lived experiences and supporting one another through what can be a profound and emotionally complex introduction to the anatomy lab. Team‐based learning in anatomy has been found to improve students' attitudes about teamwork and working with peers and is often viewed positively by students.^33^, ^34^ Additionally, having experienced students lead introductory sessions and share anecdotes helps to assure, guide, and support the students experiencing the anatomy lab for the first time, while building a sense of community.^35^ Instructors noted the mindfulness and compassion that learners displayed as they navigated the space together, often intuitively recognizing when peers needed encouragement or space and responding with care. Ultimately, the small group design of the donor meeting session fosters a sense of support and humanism in the anatomy lab, both crucial for building the peer support required in trauma‐informed learning environments.

This peer‐to‐peer support created a sense of community and shared responsibility that extended beyond the session itself. The design of the session also modeled collaboration at the institutional level. A diverse instructional team, comprising instructors, medical ethicists, and registered counselors, was present to guide, listen, and respond. The presence of this interdisciplinary group contributed to a sense of collective care and emphasized that the emotional and ethical dimensions of anatomy education are shared responsibilities. Learners were able to see that care, reflection, and humanism are not add‐ons to scientific rigor, but integral to it. This environment nurtured a collaborative culture where learners could engage authentically, feel supported by both peers and professionals, and begin to internalize the values of relational, ethical practice from the very first lab experience.

### Empowerment

Empowerment in trauma‐informed educational settings means that “individuals' strengths and experiences are recognized and built upon” and that they are supported in cultivating self‐advocacy skills.^19^ Similar to how Shiozawa and colleagues helped develop students' emotional resilience and coping strategies to use in their future careers with their seminars, our donor meeting session also embedded student empowerment in its design and delivery.^24^ The small groups, teaching assistants, and explicit time for reflection in‐lab emphasized learner agency, collaboration, and the development of professional competencies. Learners in our anatomy labs are expected to navigate the experience with a high level of independence; they are supported in their autonomy to engage with as much or as little with donors in these initial sessions and safely exit the room at any point as necessary. Indeed, during these sessions, instructors observed a wide variety of interactions and engagement—those who step back and observe (or look away), and those who move closer, probing and asking questions. All responses are normalized.

From the outset, students are encouraged to take responsibility for working through lab outlines, assigning roles within their teams, and advocating for their own needs, whether emotional, logistical, or educational. The donor meeting session was intentionally structured to support this by helping learners acclimate to the space, build comfort with the anatomical concepts, and feel confident in their ability to engage fully in their lab work. Rather than being passive recipients of knowledge, learners are positioned as active participants, capable of negotiating group dynamics, articulating strengths and limitations, and contributing meaningfully to their teams. This early empowerment sets the tone for the remainder of the program and affirms learners' capacity to manage the intellectual, emotional, and interpersonal demands of anatomy education.

### Inclusivity

Inclusivity in a trauma‐informed approach involves attending to cultural, historical, and gender issues, with an emphasis on moving beyond stereotypes and biases to foster equity, representation, and humanism in learning spaces.^19^ In our donor meeting session, inclusivity is cultivated by recognizing the diverse identities of both the donors and the learners, and by actively working to humanize those who are often objectified in traditional anatomy education. Rather than beginning with dissection of the back, where learners may not see the donor's face for weeks, we begin with an intentional introduction to the donor's whole identity, including the face and genitals. When available, we share personal details such as the donor's name, cultural background, lived experiences, medical history, and gender pronouns as provided by their families. The instructional team also makes certain to facilitate the entire donor meeting service with inclusive language to foster a safe and inclusive expectation for how to discuss the human body and donors. This grounds the session in personhood and invites learners to engage with the donor as an individual who lived in the same communities they do. This approach is particularly meaningful in our context, where we teach a diverse population of learners, many of whom come from groups historically underrepresented or marginalized in the medical curriculum.

By highlighting that donors are members of our local community, we offer moments where learners may recognize elements of their own identities in the donors, whether through cultural background, gender identity, language, or life experiences. This intentionality to bring awareness to the different aspects of the donors also aligns with student perceptions that anatomy educators should be focusing on inclusivity in anatomical education.^36^ Even if learners do not relate to all aspects of a donor's identity, the opportunity to connect with any part of it fosters a deeper sense of humanism and respect. These connections help learners reframe the donor as their first patient, shaping a relational and inclusive approach to care.^37^ Just as we honor the diverse experiences of the donors, we recognize that learners and instructors bring their own lived experiences into the lab. Creating space for these identities enhances relational learning and fosters an environment where all individuals feel seen, valued, and respected.

This trauma‐informed framework offers a cohesive lens through which the donor meeting session can be understood as both emotionally supportive and pedagogically rigorous. By integrating emotional learning with clinical preparation, it helps learners cultivate habits of respect, empathy, and reflection that extend beyond the anatomy lab into their future clinical practice. Unlike other approaches that emphasize these elements in isolation, our model weaves humanism, empathy, support, and reflection into a unified experience. In doing so, it not only strengthens resilience and professional identity formation but also fosters an environment where learners can engage deeply and meaningfully with foundational anatomical knowledge. Grounded in the key principles discussed above and supported by student and educator feedback, we recommend that this trauma‐informed framework be prioritized within health professions curricula. At a time when dedicated hours for anatomy are declining across medical education,^38^, ^39^ it is essential to safeguard time for introductory sessions that prepare learners not only for anatomical study but also for the emotional, humanistic, and professional dimensions of working with donors. These sessions equip students with tools to navigate both the anatomical curriculum and the hidden curriculum, supporting professional identity formation and emotional well‐being. Importantly, this model extends beyond medical programs and can be adapted for any discipline that learns from donors in gross anatomy laboratories. Rather than optional additions, these sessions should be recognized as critical components of contemporary anatomy education.

## LESSONS LEARNED

Reflecting on our experiences implementing the donor meeting sessions, and drawing from learner feedback through postsession reflections, several key lessons have been found that will guide future iterations of this initiative.

First, we identified the need to provide learners with more detailed and advanced information about the donor meeting session prior to its delivery. Entering the gross anatomy lab for the first time can be a significant emotional and cognitive experience, and allowing learners time to mentally prepare is critical. Clear communication about the structure, purpose, and expectations of the session supports learners' ability to engage meaningfully and with less anxiety.

Second, we have recognized the value of conducting the session in the smallest group sizes possible, ideally around six participants. The small‐group setting is essential for both practical operations and for fostering meaningful emotional engagement, which in turn helps build a sense of community among students. Instructors should also note that smaller groups reduce the sensory and emotional burden that can arise in a crowded lab, while providing a more intimate, personalized, and supportive experience. This approach is intended to minimize feelings of being overwhelmed and to maximize opportunities for connection with the donor, the learning environment, and with one another. While this article focuses on our undergraduate medical education context, we note the success of this session format within our other anatomical programs as well, such as the Physician Assistant (PA) program at Feinberg and the Master's of Clinical Anatomy program at Schulich. This underscores its broader relevance and suggests that this model could benefit a wider range of learners across anatomy education, such as dentistry, physiotherapy, occupational therapy, and undergraduate education.

Ultimately, it was the thoughtful design of the session that contributed to its effectiveness. Learners' open‐ended reflections affirmed that the session addressed not only their cognitive readiness for gross anatomy but also their emotional well‐being and developing professional identity. This intentional attention to the “whole learner” reinforces the value of trauma‐informed, human‐centered approaches in anatomy education.

Finally, a foundational goal of the donor meeting session was to align our curriculum with the evolving emphasis in the anatomical sciences on person‐first practices. The feedback we received from both learners and instructors confirmed that the session fostered a greater sense of humanism in the anatomy lab. These impressions are consistent with prior findings that reflective and relational approaches to dissection can enhance gratitude, connection, and positive framing of the anatomy experience.^40^ The session thus represents a meaningful pedagogical shift—one that centers care, community, and respect at the very beginning of the anatomical learning journey.

These insights emphasize the importance of intentional planning, emotional preparation, and learner‐centered design in anatomy education. As we continue to refine this session, we remain committed to fostering a respectful, inclusive, and humanistic learning environment; one that not only supports anatomical learning but also contributes meaningfully to the professional identity formation of future healthcare providers.

## CONCLUSION

In closing, the donor meeting session was designed to support learners in their first encounter with body donors and to foster a humanistic, trauma‐informed learning environment. Grounded in recommended frameworks and informed by iterative feedback, the session emphasizes safety, trust, collaboration, empowerment, and inclusivity. While we met many of the goals of the trauma‐informed principles, we acknowledge that the first iteration of this session was designed and implemented without the direct involvement of learners. Moving forward, we are committed to incorporating learner voices in the co‐creation and delivery of these sessions to further align with the principles of empowerment, collaboration, and inclusivity. At Feinberg, this process has already begun with a portion of the session now dedicated to addressing practices of body donation as they relate to marginalized groups; this portion of the session was designed and originally delivered by a student.

The overwhelmingly positive feedback from learners and instructors affirms the value of this session in building resilience, supporting reflection, and encouraging meaningful connection to the donors as the learners' first patients. Based on the literature, we are confident that instilling these professional skills early on in the anatomy lab will foster professional identity formation and equip future health care providers to carry these practices forward into their interactions with patients. Although developed within an undergraduate medical education context, this session format has proven successful across anatomical programs, highlighting its potential applicability and broad utility across diverse anatomy education settings within the health care field. As such, we recommend that anatomy educators consider how they orient learners to the gross anatomy lab and body donors, and how they might implement similar sessions to foster supportive and humanistic learning environments. While institutions will need to tailor the specifics to their local context, we offer the trauma‐informed framework as a model to guide best practices and future curricular development in anatomy education.

## AUTHOR CONTRIBUTIONS

**Bryn Bhalerao:** Writing – original draft; writing – review and editing. **Catherine Belling:** Conceptualization; writing – review and editing; resources; methodology. **Angelique N. Dueñas:** Conceptualization; writing – review and editing; methodology; project administration; resources; data curation; investigation. **Charys M. Martin:** Writing – original draft; writing – review and editing; methodology; resources; conceptualization; supervision; data curation; investigation. **Andrew M. Deweyert:** Conceptualization; writing – original draft; writing – review and editing; methodology; supervision; resources; project administration; data curation; investigation.

## CONFLICT OF INTEREST STATEMENT

The authors have no conflicts of interest to disclose.

## ETHICS STATEMENT

Ethics approval was not required for this article as it did not involve direct data collection or interaction with human participants (see https://ethics.gc.ca/eng/tcps2‐eptc2_2018_chapter2‐chapitre2.html). We received approval for QA/QI/PE (Project ID: 125571), and OHRE determined that this study does not require oversight by one of Western University's REBs on July 10, 2024.

## Data Availability

As this manuscript is a discursive article, there are no datasets associated with this paper. Therefore, there are no other papers (in progress, under review, or published) using the same dataset to disclose.
